# Elimination diets’ efficacy and mechanisms in attention deficit hyperactivity disorder and autism spectrum disorder

**DOI:** 10.1007/s00787-017-0959-1

**Published:** 2017-02-11

**Authors:** Verena Ly, Marco Bottelier, Pieter J. Hoekstra, Alejandro Arias Vasquez, Jan K. Buitelaar, Nanda N. Rommelse

**Affiliations:** 1 Karakter, Child and Adolescents Psychiatry, Reinier Postlaan 12, 6525 GC Nijmegen, The Netherlands; 2 Department of Cognitive Neuroscience and Donders Institute for Brain, Cognition and Behaviour, Radboud University Medical Centre, Nijmegen, The Netherlands; 3 Department of Psychiatry and Donders Institute for Brain, Cognition and Behaviour, Radboud University Medical Centre, Nijmegen, The Netherlands; 4 Department of Human Genetics and Donders Institute for Brain, Cognition and Behaviour, Radboud University Medical Centre, Nijmegen, The Netherlands; 5 Department of Child and Adolescent Psychiatry, University of Groningen, University Medical Center Groningen, Groningen, The Netherlands; 6 Triversum, Child and Adolescent Psychiatry, Alkmaar, The Netherlands; 7 Leiden University, Institute of Psychology and Leiden Institute for Brain and Cognition, Leiden, The Netherlands

**Keywords:** Elimination diet, Attention deficit/hyperactivity disorder, Autism spectrum disorder, Food sensitivity, Gut–brain interaction

## Abstract

Nutrition plays an important role in neurodevelopment. This insight has led to increasing research into the efficacy of nutrition-related interventions for treating neurodevelopmental disorders. This review discusses an elimination diet as a treatment for attention deficit hyperactivity disorder and autism spectrum disorder, with a focus on the efficacy of the food additives exclusion diet, gluten-free/casein-free diet and oligoantigenic diet. Furthermore, we discuss the potential mechanisms of elimination diets’ effects in these neurodevelopmental disorders. The main candidate mechanism is the microbiome–gut–brain axis possibly involving complex interactions between multiple systems, including the metabolic, immune, endocrine, and neural system. We conclude with practical implications and future directions into the investigation of an elimination diet’s efficacy in the treatment of attention deficit hyperactivity disorder and autism spectrum disorder.

## Introduction

Autism spectrum disorder (ASD) and attention deficit hyperactivity disorder (ADHD) are early onset neurodevelopmental disorders that may persist into adulthood. Both ASD and ADHD are umbrella terms that cover heterogeneity of behavioral abnormalities. ADHD is characterized by inattentive, hyperactive and impulsive behavior, whereas the key symptoms of ASD include social deficits, communication deficits and stereotypical behavior [1]. Despite the differences in the core behavioral symptoms between ASD and ADHD, there are indications for an overlap between the disorders. Indeed, research indicates that these disorders are highly comorbid [2–7]. This overlap could be explained by a shared etiology between ADHD and ASD disorders; for instance, genetic studies have demonstrated common genetic etiology for these disorders [8, 9]. Although the etiology of ASD and ADHD remains largely unknown, a complex interaction of genetic and environmental factors is thought to contribute to the development of ASD and ADHD [2, 10, 11].

One of the potential environmental risk factors for neurodevelopmental disorders is diet [12]. Nutrition has an impact on neurodevelopment, cognition, and behavior, and could therefore play an important role in neurodevelopmental disorders [13–15]. This insight, together with the lack of effective treatments for core ASD symptoms and the concerns about the safety of pharmacological treatments in ADHD [16], have led to increasing research into the efficacy of nutrition-related interventions as additional or alternative (non-pharmacological) treatments for these disorders. The dietary interventions that have been subjected to clinical trials include various forms of elimination diets and supplementation interventions [17–20]. In this review, we provide an overview of the literature with regard to the most common forms of elimination diets and their efficacy in ADHD and ASD. Furthermore, we discuss the potential mechanisms of elimination diets’ effects in both ADHD and ASD. Finally, we conclude with the practical implications and future directions into the investigation of elimination diets’ efficacy in the treatment of these early onset neurodevelopmental disorders.

## Elimination diets in ASD and ADHD

At first, the concept of an elimination diet was introduced to diagnose and treat food allergies [21]. The main reason for applying an elimination diet is to find out which foods are causing or aggravating physical adverse reactions. Later this link of food with adverse physical adverse reactions has been extended to a connection with neurobehavioral symptoms [22, 23]. Thus, in this later view, behavioral reactions are seen as possible adverse reactions to food as well. In the domain of psychiatry, it is therefore believed that elimination diets can be used to identify foods that contribute to mental disorders and associated behavioral and cognitive disturbances.

Elimination diets come in different forms and vary in their strictness and food items that are being eliminated. Interestingly, the forms of elimination diets that are being investigated differ between ASD versus ADHD. In ASD, the gluten-free and/or casein-free (GFCF) diet has been mainly investigated, whereas in ADHD, clinical trials examined the effects of food additives exclusion diets and the oligoantigentic diets. These differences in focus between the elimination diets that are applied in the two disorders are due to the different origins of the research in both fields. In the following sections, the origins, rationale, and the general principles of the diets are being described, followed by the degree of evidence with regard to the efficacy. In Table 1, an overview is provided for several characteristics of the respective diets, including the excluded food items, main target group, efficacy, and nutritional elements that would require attention in case of long-term exclusion to avoid deficiencies.

**Table 1:** Overview of the characteristics of the respective elimination diets

Diet	Exclude	Main target	Meta-analyses	Conclusion efficacy	Risk nutritional deficiencies*
Gluten-free casein-free	Gluten and casein (e.g., products containing wheat, oats, barley, or rye; and milk and dairy products	ASD	No meta-analyses available (recent extensive review by Elder et al. [19])	Results are inconclusive Weak evidence for any positive effects A subset of individuals may respond positively (e.g., those with gastrointestinal abnormalities)	Gluten exclusion: vitamin B, iron, fiber Casein exclusion: calcium, vitamin D, protein Some evidence for suboptimal bone development [135]
Food additives exclusion	Artificial food coloring Artificial flavors Artificial fragrances Preservatives Artificial sweeteners	ADHD	Two recent meta-analyses [51, 18]	Small effects Effects may not be specific to ADHD Effects may depend on food sensitivity	No risk for nutritional deficiencies
Oligoantigenic	Antigenic foods (basic diet may be restricted to a few hypoallergenic foods: turkey, pears, rice, lettuce, water)	ADHD	Two recent meta-analyses [51, 18]	Large effects including studies using proximal assessment, but small effects based on probably blinded assessment studies No information on long-term effectiveness	Vitamins, minerals, fiber, proteins No direct evidence for these risk

* Nutritional deficiencies can only occur after long-term exclusion of food products. In case of long-term exclusion, nutrient supplementation is usually recommended by the dietician. Therefore, close supervision by a dietician is important when applying these diets

### Gluten-free diet and/or casein-free diet in ASD

The focus on GFCF diets in ASD originates from the evidence of comorbid gastrointestinal tract problems in patients with ASD [24–26]. The explanation for increased gastrointestinal problems in ASD remains unclear. One explanation is that gastrointestinal tract problems in patients with ASD might be caused by increased intestinal permeability, the so-called leaky gut, which has indeed been demonstrated in children with ASD compared to healthy controls [27–29]. According to the ‘opioid excess theory’, digestion of gluten and casein produces peptides with an opioid activity that can enter the bloodstream when the gut permeability is high [30, 31]. These neuroactive peptides can in turn bind to opioid receptors and are therefore speculated to affect processes in the central nervous system [32].

The idea for the gluten-free diet involves examination of the effects of removing all the food items containing gluten, a mixture of proteins found in wheat, oats, barley, or rye. Thus, all products made with these cereals are excluded from the diet and replaced with special gluten-free version of the common food items. In some cases, the gluten-free diet is combined with a casein-free diet. Casein is a peptide commonly found in milk. Thus, a casein-free diet involves avoiding the intake of milk and dairy products. After a period of elimination, products containing gluten or casein can be reintroduced to test whether it is contributing to the symptoms.

### Food additives exclusion diet in ADHD

As discussed above, the elimination diet was originally proposed as a tool for treatment of food allergies [21]. Feingold, an American pediatrician and allergist, was the first who suggested that allergy to food additives, such as artificial flavors and colors, as well as naturally occurring salicylates could lead to ADHD symptoms [33]. He based this on observations that aspirin, which contains salicylates, could not only lead to allergic reactions, but also to an increase of hyperkinetic behavior in some patients. This observation has set the stage for the development of various food additives exclusion diets, which typically involves the removal of, for instance, artificial food colors, flavors, fragrances, preservatives, and sweeteners. Sometimes, this diet is part of a broader elimination diet, such as the Feingold diet or Kaiser Permanente diet (http://feingold.org/), which involves the removal of both natural and artificial salicylates. The intervention usually investigates the effect over periods of a week or longer. Food challenges that include food additives can be used after this period to examine the acute immediate effects of the challenge. The hypothesis is that ADHD could be influenced by either allergenic or pharmacologic mechanisms related to levels of salicylates and salicylates-like substances.

### Oligoantigenic diet in ADHD

In oligoantigenic diets, the focus is on eliminating suspected high allergenic food products rather than eliminating artificial colors, flavors, and preservatives specifically. An oligoantigenic diet intervention, also known as a restricted elimination diet or hypoallergenic diet, involves the testin657g of an individually constructed restricted elimination diet. The focus is on eliminating foods that are often found to be highly allergenic, such as cow’s milk, cheese, egg, chocolate, and nuts. Oligoantigenic diets come in different forms and vary in their strictness. The oligoantigenic diet typically involves an elimination phase (usually two to five weeks) in which the specific food items are excluded completely. The food items in the elimination phase could, for instance, consist of only a few hypoallergenic foods such as rice, turkey, lettuce, pears, and water [34]. If the patient reacts by a substantial decrease of symptoms indicating ‘food sensitivity’, a reintroduction phase, which could take as long as eighteen months, could be applied to find out what specific food items trigger the symptoms. Thus, an elimination diet can be regarded as a ‘diagnostic’ tool for determining whether specific foods cause adverse physical and/or behavioral reactions.

## Evidence for elimination diets’ effects on ADHD and ASD symptoms

### Gluten-free/casein-free diet in children with ASD

There is increasing research into the effectiveness of the GFCF diet in children diagnosed with ASD. Several systematic reviews of GFCF diet studies in ASD have appeared [19, 35–40]. However, the number of high quality RCTs is too small for drawing firm conclusions at this point and there are no meta-analyses available. Overall, the findings with regard to GFCF diet’s effect in ASD are mixed. Several studies suggest no effect of the GFCF diet’s effect in ASD [41–43]. For instance, a recent study testing the effects of a GFCF diet in children with ASD (*n* = 30) involving a double-blind placebo-controlled challenge phase did not demonstrate positive effects on measures of physiological functioning, behavior problems, or ASD symptoms [41]. However, children with known gastrointestinal disorder were excluded, which could have weakened any potential effects [41]. In fact, there is research suggesting that gastrointestinal issues might go together with ASD behavioral symptoms and reactions to gluten and casein [46, 47]. Two RCTs investigated GFCF diet’s effect in ASD by following children with ASD (*n* = 10 and *n* = 72) for 1 year [44, 45]. The data demonstrated positive effects of the GFCF diet on ASD trait measures. However, the outcome measure was based on unblinded caregivers’ reports and the studies did not control for concomitant treatments that the children may have received while being enrolled in the studies. Furthermore, the attrition rate was nearly a quarter in one of the studies, which was not taken into account in the analysis.

### Elimination diets in children with ADHD

The effectiveness of the food additives exclusion diet as treatment for ADHD has been investigated in a number of studies [17, 18, 48]. Only some studies additionally eliminated food items containing natural salicylates as part of a broader diet, such as the Feingold diet or Kaiser Permanente diet [49, 50]. A meta-analysis with a focus on the effectiveness of such elimination diets in ADHD across twenty studies including 794 participants found a small effect size based on parent reports, 0.18, that decreased to 0.12 when taking into account possible publication bias [51]. The effects based on teachers’ reports and observer measures were not significant. However, pooling the data of a limited number of high quality studies for analyses in this meta-analysis demonstrated small effects on teacher ratings and neurocognitive attentional tests. In another meta-analysis, focusing exclusively on ADHD outcomes and more critically addressed assessment blinding issues, eight studies including 294 participants reported an effect size of 0.32 for the most proximal assessment and 0.42 for the probably blinded assessments [18]. It is important to note though that the effects of this meta-analysis may be limited to children with ADHD who have sensitivity to food colors, as the participants were often preselected on the basis of a suspected sensitivity to food colors during the elimination phase of the diet. Furthermore, rather than an increase in the effect size, additional analysis using only trials with low/no comedication to exclude the impact of effective medication led to a reduction in the effect size to a non-significant level [18]. Stevenson et al. reported that high quality studies showed an effect size of 0.21 and 0.22 of food color elimination; however these studies were not restricted to children diagnosed with ADHD [17]. It has been suggested that the effect of food color elimination on behavior is probably not limited to children with a diagnosis of ADHD, but rather also applies to hyperactive behavior in children more generally [48].

With regard to oligoantigenic diets, the effectiveness of the elimination phase has been demonstrated in several randomized clinical trials with patients with ADHD. An effect size of 0.29 has been reported in a meta-analysis across six controlled trials including 195 participants; it was concluded that about one third of the children with ADHD show an excellent (>40% symptom reduction) response [51]. This meta-analysis excluded two studies by Pelsser et al. with an outlier effect size and the use of non-blinded ratings [34, 52]. Another meta-analysis including these two studies estimated an effect size of 1.48 that, however, dropped substantially to 0.51 (95% confidence interval −0.02 to 1.04) when probably blinded raters were used [18].

Taken together, it remains inconclusive whether elimination diets are effective as a treatment for children with ASD and ADHD. So far, the evidence for GFCF diets as an effective treatment for children with ASD is weak, but it might be a beneficial intervention for a subset of children with ASD, particularly for those with comorbid gastrointestinal problems. The observed effects for food additives exclusion diets in children with ADHD are small, and may not be specific for children with ADHD. Although a few select studies using most proximal assessment demonstrated large effects of oligoantigenic diets in children with ADHD [34, 52], an overall small effect of this diet was shown by other studies using probably blinded assessments. Furthermore, it remains unknown whether children will have a consolidated diet after the elimination phase of the oligoantigenic diet. This question calls for studies that include food challenges or a reintroduction phase in the study design. Double-blind placebo-controlled challenges or reintroduction phases after positive reactions to eliminations are useful for further investigations to test the effects of the food items in a systematic and controlled way. Studies that assess the long-term effectiveness are necessary to draw conclusions about elimination diet’s effects in children with ADHD and ASD. Finally, contrary to most aforementioned work, future studies should adopt the gold standard for clinical trials, i.e., high quality randomized controlled trials with blinded raters for the primary outcome.

## Mechanisms of elimination diets’ effect

### Food allergy and hypersensitivity

A typical food allergic reaction is mediated by specific immunoglobulin (Ig) antibodies, IgE. Such IgE-mediated allergic reaction has an immediate onset of symptoms within several minutes to several hours after contact with the allergen. The dietary interventions in children with ASD have mainly focused on the elimination of gluten and/or casein and were based on the hypothesis that children with ASD have an increased risk of immune responses to these substances. Although no association between gluten sensitivity and ASD has been reported based on immune markers [24], serum levels of immunoglobulin antibodies IgA, IgG, and IgM specific for milk-derived allergens and total IgE were increased in children with ASD compared with controls [53]. In addition, stimulation of peripheral blood mononuclear cells of children with ASD using milk-derived allergens produced more pro-inflammatory cytokines compared to controls [54]. Overall, there is increasing evidence that dysregulation of the immune system plays an important role in ASD. For instance, it has been shown that children with ASD have reduced regulatory T cell response, indicating reduced tolerance to antigens in these children [55]. ASD has also been associated with increased levels of pro-inflammatory cytokines in the blood, and decreased levels of anti-inflammatory cytokines [56, 57]. Furthermore, deficiencies in the complement system, which plays an important role in promoting immune cells to clear microbes, infected cells, and cellular debris, have also been demonstrated in ASD [58, 59]. Taken together, there is some indication that ASD is linked to food allergy or ‘hypersensitivity’, which could provide an explanation for the potential efficacy of GFCS diets in the treatment of ASD.

The aim of food additives exclusion diet and oligoantigenic diet is to eliminate foods from the diet that trigger adverse physical, allergic reactions. Thus, the concept of these diets is based on a link between ADHD and allergic reactions to food. It has been shown that children with ADHD have an increased risk of developing allergies and vice versa [60, 61]. However, so far there is no convincing evidence for food allergy or hypersensitivity to be involved in ADHD. For instance, no differences were found between patients with ADHD and healthy controls on a skin prick test to food allergens [62]. Moreover, hyperactive children who responded to a challenge test with allergenic food products did not demonstrate positive skin prick tests [63]. These findings may suggest that ADHD is associated with a non-IgE-mediated reaction to food. It has been hypothesized that determination of IgG may be helpful in these cases, such that foods that induce high IgG-levels would lead to a worsening of symptoms, while foods that induce low IgG-levels would not. However, there is no evidence for IgG as a potential marker at this point. In a previous study, a challenge test with allergenic foods has led to relapse of ADHD symptoms independently of influences on IgG blood levels, thus questioning the role of IgG-mediated mechanisms [34]. Despite the lack of clear serological markers indicating food allergy in ADHD, future studies should further investigate this open question and examine whether food allergy or hypersensitivity could play a role in ADHD and mediate elimination diets’ effects, particularly given the previous findings suggesting a link between ADHD and allergies [60, 61, 64].

### Microbiome–gut–brain axis

#### Diet and the role of the microbiome in health and disease

The intestinal microbial flora is involved in appetite regulation, energy utilization, digestion and absorption of nutrients [65, 66]. Additionally, our microbiome plays a crucial role in health and disease by influencing immune function, drug metabolism and protection against pathogens [67–69]. Disruption of the microbiome composition or dysbiosis has been associated with several human diseases, including inflammatory bowel diseases, cancer, obesity, metabolic syndrome and neurological disorders [70–77]. Moreover, the role of microbiome in multiple complex disorders is supported by evidence from safe treatment using fecal transplants in inflammatory bowel diseases, but also in disorders less directly linked to the gut, like multiple sclerosis and chronic fatigue syndrome [78]. Current evidence from animal studies convincingly shows that the composition of the microbiome has an impact that is even beyond disease, (co-)determining mood, stress response, and several aspects of behavior [79–82]. Diet is an important determinant of gut microbiota composition and functioning that is strongly linked with psychopathological outcomes [83–85]. Together, these findings suggest that there is a link between diet, the composition of the gut microbiome and mental disorders.

#### Multiple pathways in the microbiome–gut–brain axis

The effects of the microbiome on brain function, behavior and diseases can be mediated through different pathways of the so-called microbiome–gut–brain axis, involving neural, as well as metabolic, immune and endocrine mechanisms [79, 86–88]. A number of reviews have provided a thorough overview of these complex interactions [79, 86–88]. In brief, at least three pathways have been suggested to link the gut microbiome with brain function. First, the brain and the intestinal system are connected via the vagus nerve, a major nerve of the parasympathetic nervous system. The microbiome could influence brain function by innervation of the vagus nerve. The vagus nerve seems to differentiate between non-pathogenic and pathogenic bacteria and mediate signals that can induce anxiogenic and anxiolytic effects depending on the nature of the stimulus. These effects potentially involve immunomodulatory mechanisms [89]. For example, in a rodent study, it has been shown that ingestion of the *Lactobacillus rhamnosus* reduced stress-induced corticosterone and anxiety- and depression-related behavior [90]. These effects were accompanied by alterations in central γ-aminobutyric acid (GABA) receptor expression, which has often been associated with anxiety, depression, and bowel disorders [91]. Crucially, these neurochemical and behavioral effects were not found in vagotomized mice, suggesting that the vagus nerve plays a mediating role in these effects and functions as a communication pathway between gut bacteria and the brain [90]. Second, the microbiome could influence brain function via interactions with the immune system [92, 93]. Bacterial species can affect the immune system effects through the production of immune-regulatory metabolites, such as short-chain fatty acids (SCFAs) [94–96]. Alterations in regulation of neuroactive bacterial products or metabolites, can alter gene expression, influence the immune system, and interact with nerve cells by stimulating the sympathetic nervous system [97–99]. Third, the composition of the microbiome might influence the levels of neurotransmitters and thereby processes in the brain. For instance, bacterial strains can influence the synthesis and thereby the release of serotonin, a key neurotransmitter in the gut–brain connection that plays an important role in gastrointestinal and neurobehavioral regulations [100–102]. A recent rodent study has shown evidence that indigenous spore forming bacteria are able to stimulate serotonin-producing colonic enterochromaffin cells and modulate gastrointestinal motility via metabolic signaling with specific metabolites. Furthermore, an increase of the particular metabolites was associated with increases in colonic and serum serotonin in the mice, suggesting an important role of the spore forming bacteria in the regulation of serotonin levels and serotonin-related processes in the host. In addition to serotonin and SCFAs, gut bacteria are also capable of producing an array of other neuroactive and immunomodulatory compounds, including dopamine, GABA, histamine, and acetylcholine [103–106].

#### The role of the microbiome–gut–brain axis in neurodevelopment

The development of the microbiome–gut–brain axis happens early in life. The bacterial colonization is a postnatal event, which commences at birth. After one-to-three years of age, a complex adult-like microbiome is evident and stable [107–109]. Parallels can be drawn between the development of the microbiome and the development of the central nervous system suggesting that critical windows in neurodevelopment might be linked to critical periods in the colonization of the microbiome [86]. It has therefore been suggested that the microbiome–gut–brain axis plays an important role in neurodevelopmental disorders [86, 88]. However, the precise factors in the microbial composition that may cause disruptions in neurodevelopmental processes are not yet identified. Nevertheless, there is some evidence in support for this idea: for instance, it has been shown that germ-free mice have an exaggerated HPA-axis response to an external stressor, while this effect was reversed by administration of the feces from specific pathogen-free mice with normal gut microbiota at early stage of development, but not at later stage [110]. This suggests that exposure to endogenous microbiota at an early developmental stage is required for the HPA system to become fully susceptible to inhibitory neural regulation. More generally, these findings suggest that the microbiome may contribute to critical windows in neurodevelopment, and it is relevant to consider the role of the microbiome in neurodevelopmental disorders, such as ASD and ADHD.

#### The role of the microbiome–gut–brain axis in ASD

In individuals with ASD, altered composition of the intestinal microbiota has been demonstrated [111–115]. For instance, studies in ASD have found *Clostridium* or *Desulfovibrio* bacteria clusters over-represented in children with gastrointestinal complaints and ASD as compared to children with similar gastrointestinal complaints but typical neurobehavioral development [113, 116, 117]. Although the results with regard to the specific species of microbes that are altered in individuals with ASD versus controls are inconsistent across studies, alterations in the *Clostridium* species have been reported across several studies in individuals with ASD [111, 113, 117]. Clinical improvement has additionally been reported anecdotally in children with ASD who develop fever [118], receive oral antibiotics [119], or ingest probiotics [120], all of which likely alter the gut microbiome. Using an animal model of ASD with gastrointestinal and neurological symptoms, the supply of *Bacteroides fragilis*, commensal intestinal bacteria, has been shown to reduce the gastrointestinal symptoms, improve the intestinal barrier permeability, and reduce ASD-related behavioral disturbances [121].

As discussed previously, other theories with regard to the mechanism underlying elimination diet’s effects in ASD relate to metabolic deficiencies (though not excluding a role of the microbiome). It has been postulated that ASD is the result of a metabolic disorder in which opioid peptides produced through metabolism of gluten and casein pass through an abnormally high permeable intestinal membrane (‘opioid excess theory’ or ‘leaky gut theory’) [27–31]. These neuroactive peptides can then enter the blood stream and exert an effect on neurotransmission through binding with opioid receptors. Indeed, a higher permeability of the gut has been found in children with ASD compared to the gut permeability of healthy controls [27]. However, so far experiments have failed to detect any abnormal high levels of opioid peptides in children with ASD [122]. Another potential theory relates to oxidative stress and sulfur metabolic deficiencies. In a vicious circle, sulfur metabolic deficiencies might influence the bacterial composition and bacterial products leading to elevated oxidative stress in individuals. Together, bacterial products, oxidative stress and dietary allergens could lead to increased intestinal permeability [123]. Neuroinflammation, affecting the protective blood–brain-barrier capacity, as a result of increased gastrointestinal permeability could contribute to ASD. Therefore, a dietary treatment that is able to restore the gut permeability can be effective as a treatment for children with ASD [123–125].

#### The role of the microbiome–gut–brain axis in ADHD

Although direct evidence for a role of changed microbiome–gut–brain interaction is currently lacking in ADHD, converging evidence suggests that similar alterations in microbiome–gut–brain interaction can contribute to ADHD symptoms as well [64, 126]. Our group has recent unpublished data showing that gut microbiome is altered in ADHD. More specifically, increases in the genus *Bifidobacterium* in ADHD versus controls contributed to the increased metabolic function of cyclohexadienyl dehydratase, an enzyme that is involved in the synthesis of the essential amino acid and dopamine precursor, phenylalanine. Furthermore, in the same study, we demonstrated that these metabolic changes were related to decreased ventral striatal signals to reward anticipation, a neural hallmark of ADHD [127, 128]. These findings could be an important first step towards a better understanding of the relationship between alterations in the microbiome–gut–brain axis and ADHD symptoms. Further investigations are necessary to establish this relationship, and the role of diet.

#### Family relationships and structure

Since strict parental supervision is necessary for the application of an elimination diet, one could argue that elimination diets’ effects might be explained by concomitant adaptations in parental behavioral strategies and care. It is known that parents of children with ADHD and ASD use different parenting structure and have a different relationship with their children [129–131]. Furthermore, consistent parenting and positive parent–child interactions are associated with improvements of child behavior [132]. Although it should be noted that for ADHD, behavioral interventions mainly have shown positive effects on other outcomes that are associated with the disorder rather than on the primary symptoms of the disorder [18, 133]. Nevertheless, one could argue that there is a possibility that elimination diets’ effects as reflected by behavioral improvements might be mediated by the change in parenting structure required for the application of the elimination diet, rather than by a direct result of dietary changes per se. However, this hypothesis has not been supported by any evidence so far. In a previous study, it was demonstrated that families of children with ADHD, who were motivated to follow an elimination diet, already showed a good family environment, i.e., no difference compared to families of health controls. Moreover, the elimination diet did not affect the family relationships and structure [134]. More research is necessary to investigate whether family relationships, family structure, and other psychosocial variables may play a potential role in elimination diet’s effects.

In sum, the mechanism underlying the effectiveness of the elimination diet is yet unknown. The microbiome–gut–brain axis is the main candidate mechanism and could potentially involve interactions between allergic reactions, intestinal permeability, oxidative stress, and alterations in microbiome composition and function. Characterization of the direct and indirect pathways of the microbiome–gut–brain connection is important, as it can ultimately inform clinicians as to potential markers and targets for (preventative) treatment. Furthermore, this knowledge acquisition is an important step in the development of novel nutritional/therapeutic interventions tailored at the individual patient. More knowledge about the mechanisms underlying elimination diets’ effects in ADHD and ASD is needed.

## Clinical implications and future research

The GFCF diet might be beneficial for children with ASD and food intolerance/allergy or underlying gastrointestinal disease. However, the evidence for the effectiveness of GFCF diets in children with ASD is weak and thus these diets cannot be generally recommended as a treatment for children with ASD. Yet, a GFCF diet is a commonly applied intervention in children with ASD in practice. Many parents may feel it is better to apply any available treatment when searching for treatment for their child with ASD as therapies for treating core symptoms of ASD are limited. However, using a GFCF diet may not be without harm (e.g., stigmatization, diversion of treatment resources, nutritional deficiency) [135]. Hence, until there is conclusive evidence for the benefits of GFCF diets for children with ASD, it is the task of health professionals and researchers to communicate with caregivers and inform them about the costs and potential adverse consequences.

Benefits of food additives exclusion diets have been suggested in children diagnosed with ADHD. However, the observed effects are small, which makes food additives exclusion diet not qualified as stand-alone treatment. Nevertheless, given the positive, albeit small effects of this intervention in children with ADHD as well as in children from the general population, and the fact that food additives do not provide any health benefits, it is recommended that children preventatively minimize consumption of processed food products with these ingredients. Moreover, from a public health perspective, food industry might change its policy and lower or even avoid adding unnecessary additives to food.

With regard to oligoantigenic diets, if applied under close supervision, these interventions could be valuable instruments to assess whether ADHD is triggered by food. However, the efficacy of this diet as a treatment for ADHD remains to be investigated as there are no studies yet that have demonstrated long-term beneficial effects of this treatment after consolidating the diet. Our research group is currently conducting a large clinical trial to assess the long-term beneficial effects of an oligoantigenic diet in children with ADHD.

Previous work has shown that only a subset of the children with ADHD responds to an oligoantigenic diet [34]. However, the characteristics of responders versus non-responders remain to be determined in future work. In the application of an elimination diet, it is important to take the individual differences in treatment response into account. First observations from our ongoing randomized controlled trial (www.project-trace.nl) suggest that some children might not improve on the primary symptoms, but rather on symptoms that usually accompany the disorder, such as emotion regulation problems (e.g., irritability) and physical symptoms (e.g., intestinal disturbances). These observations might suggest that the effects of an elimination diet are not per se symptom/diagnosis specific and it could be important to monitor changes in different domains that are relevant for the well-being of the child. Furthermore, we have observed large individual differences in terms of time that is needed for any positive effects of the diet to surface during the elimination phase. Some children appear to be ‘fast-responders’, who show a positive reaction after a few days, whereas other children are ‘slow-responders’, who show a positive reaction after a couple of weeks. It remains to be investigated what could explain these individual differences and whether these individual differences are useful indicators for optimizing the individual treatment by reducing the duration of the treatment. Similarly, individual variation during the reintroduction phase is evident, with aggravation of ADHD symptoms in some children after food reintroduction after 5 days, whereas others only respond after 10–14 days. In addition, children with little variation in their normal food habits tend to respond more strongly to the intervention, but also seem to have the most difficulty in adhering to the diet since they dislike many foods that are present in the elimination phase. This may result in the child eating too few different foods during this phase, increasing the risk for non-adherence since the elimination phase is then overly strict. For the feasibility of this intensive treatment, it is also crucial to consider the optimal moment to start the diet. Stressors or life events, such as change of school environment or family structure, illnesses, but also positive events, such as celebrations and holidays, may negatively impact the motivation and feasibility of the successful application of the treatment. The reintroduction phase is the most intensive part of the treatment given its long duration. Close supervision by professionals when applying this diet or any other elimination diet is necessary such that effects of the diet as well as parent–child relationships can be monitored, the diet can be adjusted if needed, and training, support and guidance can be given to prevent the risk of adverse consequences. Apart from stigmatization, diversion of treatment resources, and nutritional deficiency, parent–child interaction problems may also be a risk. For instance, it has occurred that the child seized the opportunity to put pressure on parents to get what he/she desires with the ‘threat’ of not adhering to the diet. Practitioners should inform the caregivers that an elimination diet requires a change of lifestyle, and success will depend largely on the level of control over the child’s diet and behavior, and the resources and organizational capacities within the family. Finally, caregivers should be reminded that the decision to explore the effects of an elimination diet should be weighed against potential adverse consequences.

Future research is necessary to establish the clinical utility of elimination diets in the treatment of children with ADHD and ASD, particularly considering the widespread use of these treatments. Adequately powered, long-term RCTs, including blinded assessment of the primary outcome, should be conducted to determine the effectiveness and cost-effectiveness of elimination diets for children with ADHD and ASD. In these trials, a reintroduction or food challenge phase, preferably double-blind placebo-controlled, should take place after the elimination phase to determine the effects of the eliminated food items thoroughly. It remains to be investigated whether effects of specific foods can be specific for certain disorders or behavioral problems. In addition, many questions pertaining to the feasibility of these treatments remain to be answered. Attrition rates ranging from 0 to 25% have been reported in the literature, but these numbers were mostly based on a range of short-term trials with large heterogeneity in the designs (e.g., in preselection of patients, duration of trial, and nature of controlled challenge phase of the experiment). Thus, long-term clinical trials are particularly important for answering questions related to feasibility. Finally, this work should investigate how biopsychosocial variables may be affected by the treatment and may play a role in the efficacy of the treatment.

New avenues should also include the identification of predictors of treatment response, especially given that elimination diets may only improve the behavior in a (small) subset of the affected children. Future research might also target the microbiome–gut–brain connection as a potential mechanism that underlies the development of neurodevelopment disorders and elimination diets’ effect in reducing symptoms [126, 136]. A multidisciplinary approach is essential for the investigation of the relation between the microbiome–gut–brain connection and behavioral symptoms of ASD and ADHD, which potentially involves multiple systems, including the metabolic, immune, endocrine, and neural system. The knowledge acquisition pertaining to the mechanism of action could ultimately help inform clinicians about potential markers and targets for preventative and individualized treatment.
